# Comparative study on the Impact of Transcranial Magnetic stimulation and Bio-feedback on overactive bladder in multiple sclerosis patients: a Randomized Clinical Trial

**DOI:** 10.1007/s10072-024-07788-y

**Published:** 2024-10-21

**Authors:** Osama M. Abdel Raheem, Doaa A. Abdel-Hady

**Affiliations:** 1 Department of physical therapy for Neurology, Faculty of Physical Therapy, Deraya University, EL-Mina, Egypt; 2 Department of physical therapy for women’s health, Faculty of Physical Therapy, Deraya University, EL-Mina, Egypt

**Keywords:** Multiple sclerosis, Overactive bladder, Biofeedback, Transcranial magnetic stimulation, PFME, Urodynamic parameters

## Abstract

**Background:**

Overactive bladder (OAB) is a common clinical presentation in patients with multiple sclerosis.

**Objectives:**

The purpose of this study was to compare the effects of transcranial magnetic stimulation (TMS) and biofeedback on overactive bladder in patients with multiple sclerosis.

**Methods:**

This research included 45 individuals with multiple sclerosis of both sexes. We randomly divided them into three equal groups (A, B, and C). Patients in group A got biofeedback training and pelvic floor exercise (PFME); patients in group B had transcranial magnetic treatment and PFME; and patients in group C had PFME. Urodynamic measurements were utilized to determine bladder parameters (detrusor pressure at maximum flow rate, bladder volume at initial desire to empty, maximum cystometric capacity, detrusor pressure, and maximum flow rate) for all groups before and after a six-week training interval (the end of therapy).

**Results:**

There was a statistically significant improvement in all urodynamic measurement parameters within the groups (Groups A, B and C). Except for the maximal cystometric capacity and detrusor pressure were non-significant improvement in B before and after therapy. However, there was no significant difference between the three groups following therapy.

**Conclusion:**

Transcranial magnetic stimulation and biofeedback improved bladder function in patients with multiple sclerosis. These approaches have a high level of safety and effectiveness, but EMG biofeedback has superiority.

**Supplementary Information:**

The online version contains supplementary material available at 10.1007/s10072-024-07788-y.

## Introduction

Multiple sclerosis (MS), a form of demyelinating condition of the central nervous system (CNS), is an autoimmune-mediated neurodegenerative disorder identified primarily by inflammatory demyelinating symptoms [1]. Bladder dysfunction is frequent in multiple sclerosis (MS), influencing 80–100% of patients at some point through the disease’s progression [2]. Neurogenic detrusor over activity appears to result from either increased bladder afferent input or abnormal neural processing of afferent input, resulting in decreased suprapontine inhibition [3]. Overactive bladder (OAB) symptoms are present in 60–80% of patients due to hyperreflexia/neurogenic detrusor over activity (NDO). Other bladder diseases include decreased detrusor contraction in 20% of patients owing to hypotonic bladder and a lack of synchronization in 25% of patients due to detrusor-sphincter dyssinergia (DSD) [4–6], which can result in voiding dysfunctions, incomplete emptying, or urine retention. Mixed symptoms have a higher incidence than storing or voiding problems alone [7]. TMS of the motor cortex causes long-term changes in spinal cord excitability [8]. TMS is a non-invasive approach for causing depolarization or hyperpolarization in brain neurons [9]. TMS can measure cortical excitability and connection. These not only give critical MS diagnostic components but also monitor treatment-induced neural alterations. TMS may be used as a surrogate marker for MS, such as when assessing impairment. As previously stated, sTMS and pTMS are widely employed in the diagnosis of MS. sTMS may detect factors such as resting motor threshold (RMT), motor evoked potential (MEP), and central motor conduction time (CMCT). Increased RMT, extended MEP latencies, and CMCT are linked to MS impairment. Furthermore, pTMS can measure the physiological connectivity of cortical neuronal populations and pyramidal cells. pTMS is a stimulus in which two TMS pulses are delivered in pairs at a particular time period [known as the interstimulus interval (ISI)]. The initial pulse of pTMS is known as the conditioned TMS (CS), whereas the second is known as the test TMS. The most typical strategy is to do a subthreshold technique [10]. The forerunner to BF in PFM therapy, the patient constantly examines the quality of contractions using visual cues (which can also be audio). There’s also an indication and a range for typical muscular tension. Throughout treatment, the individual has to tighten their muscles in such a manner that the monitor reading is near the physiological range of contraction [11].

To date, studies have shown great tolerance as well as objective improvement in OAB symptoms through using biofeedback and PFM training or managing with TMS and PFME and comparing the effectiveness of PFMT, biofeedback and repetitive transcranial magnetic stimulation (rTMS) as adjunct treatments for neurogenic bladder dysfunction in spinal cord injury patients. But no comparison between TMS and biofeedback on OAB in MS patients has been found. The purpose of the trial is to compare the effects of TMS stimulation and biofeedback on overactive bladder patients with MS, which could assist in the creation of a physical therapy plan for the treatment of OAB patients with MS.

## Materials and methods

1. Study Design
  The research was designed as a randomized, blinded controlled trial. It was approved by the Institutional Review Board and Deraya Ethics Committee (No. 21/2023), approval date: 1 March 2023. Every participant gave written consent before participation. The study followed the Helsinki Declaration Guidelines for the Conduct of Human Research. It lasted from March 1, 2023, to September 30, 2023.
2. Eligibility criteria
  All cases were recruited after neurological and urological referrals; 45 patients with MS and OAB were chosen at random from a physical therapy outpatient clinic. Their ages varied from 25 to 45 years, and their BMI was 25–30 kg/m2. They were randomly assigned to one of three equal groups (A, B, and C). Patients in group A received EMG biofeedback training and PFME; patients in group B received TMS and PFME; and patients in group C received PFME. Before and after a six-week training interval (the end of therapy), urodynamic measurements were used to determine bladder parameters (detrusor pressure at maximum flow rate, bladder volume at initial desire to empty, maximum cystometric capacity, detrusor pressure, and maximum flow rate) for all groups.
  Inclusion Criteria:
    Participants included both female and male MS patients ranging in age from 25 to 45 years, with BMIs ranging from 25 to 30 kg/m2. OAB diagnosis based on neurological and urological findings. These individuals have stable clinical MS. Clinical stability can be characterized as the lack of aggravation or deterioration in the Expanded Disability Status Score (EDSS) score in the six months prior to study admission.
  Exclusion Criteria:
    MS, a serious impairment, was among the exclusion criteria. Seizures, urethral slings, bladder/bladder neck suspension surgeries, prior bladder repair procedures, being pregnant or intending to become pregnant, patients with cardiovascular illness, auditory or visual impairment, intracranial pressure, pacemaker, tricyclic antidepressants, Use drugs that affect urination during treatment and other neurological problems in addition to MS.
3. Randomization and masking
  All patients were told about the nature, purpose, and benefits of the study, as well as their freedom to decline or withdraw at any time and the confidentiality of any collected data. A computer program executed the randomized operation in blocks with a 1:1:1 allocation ratio. The study numbers (one for biofeedback and PFME, two for TMS and PFME, and three for PFME) were placed in opaque safety envelopes, which study participants unsealed after filling out the consent form and receiving their first evaluation in the first session. The patients were split into three categories: A, B, and C. Blinding is maintained throughout the trial, with the researchers responsible for data processing, and participants unaware of the group assignment, The statisticians and outcome assessors were not given any information on the patients (Fig. 1).
  **Fig. 1:**
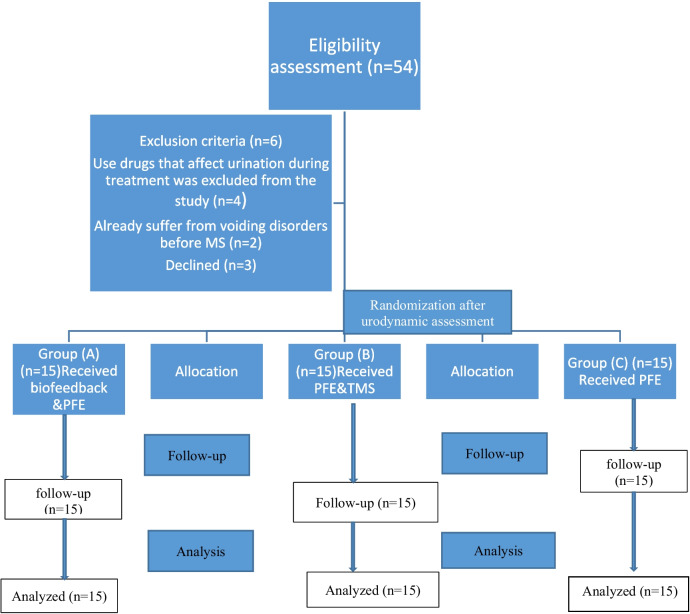
Flow chart of the trial
4. Outcome measures
  A multi-lumen urodynamic catheter is introduced into the bladder while the patient is supine. This specialized catheter is typically 6–7 ft. in diameter and constructed of polyvinyl chloride or polyurethane. It features numerous lumens for simultaneous pressure monitoring and fluid infusion. The catheter is inserted into the bladder carefully using lubricating gel containing local anesthetic. To assess abdominal pressure, a second catheter is inserted into the rectum or vagina. Other cavities to consider are an intestinal stoma and the stomach. These catheters are taped to the patient to prevent accidental movement and evacuation [12]. Urodynamic Dantic 5000/5500. Urodynamic investigation equipment, such as voiding cystometry, was used to do the urodynamic examination. The urodynamic machine is a trolley-mounted appliance with an integrated printer and display. The urologist measured the participants both before and after the trial (after six weeks). Urodynamic studies allow for a more precise assessment of the pathophysiology of urine dysfunction and risk factors for urinary tract damage in MS patients, assisting in the planning of their appropriate care. Indeed, diagnosing urinary dysfunction is difficult due to the complexities of pathophysiologic variables. Thus, urodynamic investigations are required in many situations to better understand the pathogenesis of symptoms and select the appropriate therapeutic methods. Urodynamic measures were utilized to evaluate bladder parameters (detrusor pressure at maximal flow rate, bladder volume). Bladder volume at initial desire to empty, maximum cystometric capacity, detrusor pressure, and maximum flow rate) [13].
5. Therapeutic procedures
  The therapy used in all groups consisted of three sessions per week for six weeks.
  A. Biofeedback training
    Group A received biofeedback training in the following manner: using a Myo 200 EMG biofeedback device, and before beginning the therapy sessions, all participants were requested to empty their bladders to ensure that the patients were relaxed and comfortable in Crock posture. Before the start of the training to secure the insertion of surface electrodes and reduce resistance. It was also washed with alcohol before electrode installation to reduce skin impedance. After soaking in a 1% saline solution, the earth electrode was attached to the knee. The positive electrode was on the perineum area, while the negative electrode was approximately 3 cm later on the bulk of the PFM. The findings were then saved in the same file for each patient. After preparation, the program of biofeedback. The patient was taught to produce a strong muscle contraction, after which the EMG activity during maximal contraction was recorded, and the device provided audible feedback to the patient. The physical therapist encouraged the patient to make every attempt to improve the auditory feedback signals. Activation to maximum contraction was maintained for a 15–30 s hold and a gradual release with a 15–30 s rest period. The exercise sessions were done three times a week for 30 min each.
  B Transcranial magnetic stimulation
    Group B received transcranial magnetic stimulation in the following:
    The TMS coil is positioned on the outer layer of the cerebral cortex, and the magnetic field it produces induces an electrical current in the interneurons situated in the appropriate region of the brain, leading to the neurons to release an excitatory postsynaptic potential that sends nerve impulses to the governing organs [14]. The TMS machine is outfitted with figure-of-eight coils. To maintain consistency throughout sessions, the stimulation site was marked with vertex-centered coordinates as a reference point. The coil was set at the transverse plane, with the handle pointing laterally to create lateral-to-medial current flow in the cortex [15]. Patients were given information about the device and its effects. All patients were informed about the application process. The patient would sit on a chair with suitable heights, all program parameters were utilized. 30-minute treatment time with 2 gauges of intensity, 1 Hz frequency (Fig. 2**).**
    **Fig. 2:**
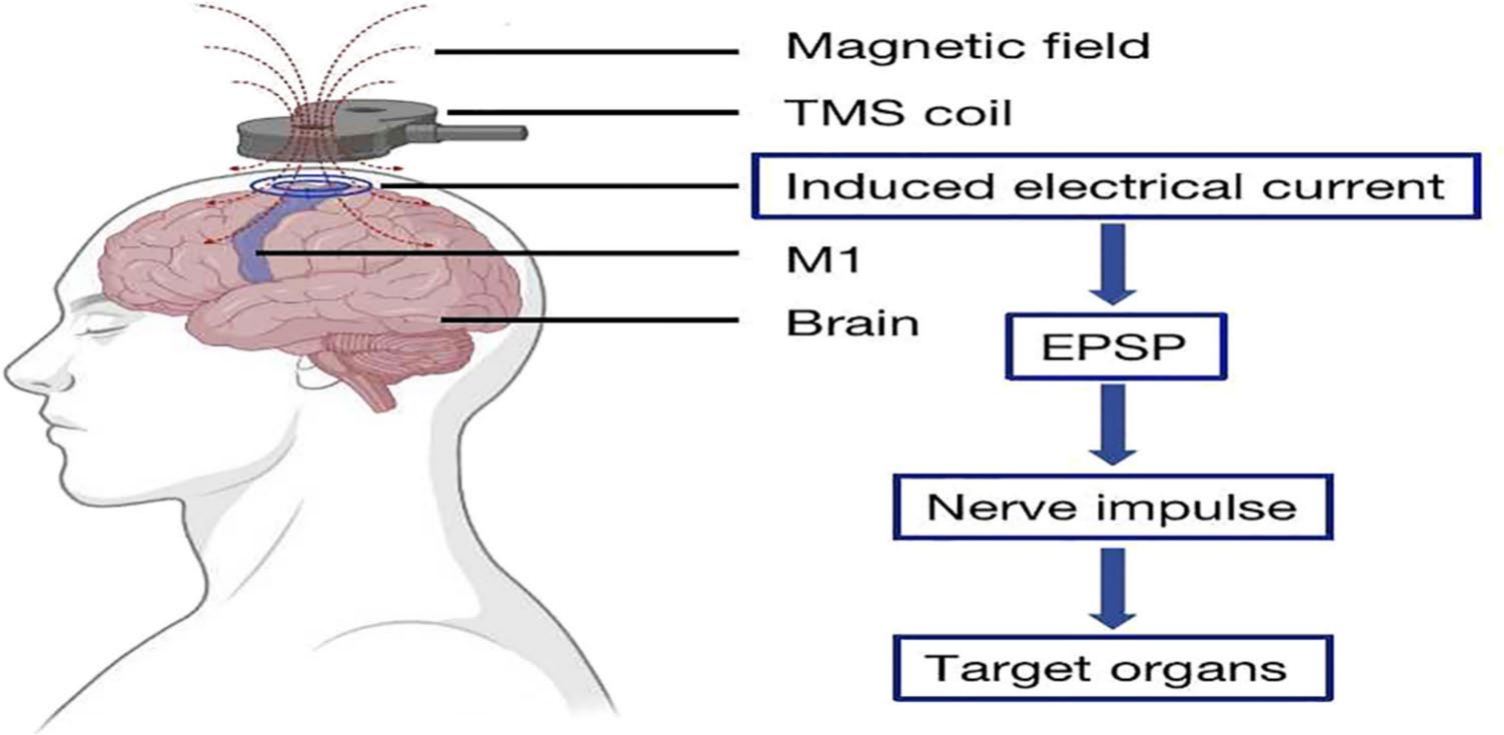
Transcranial magnetic stimulation (TMS) principle [14]
  C Pelvic floor exercises (PFME)
    Group A & B and C received PFME
    - All patients in group C were asked to evacuate their bladder before starting the treatment sessions to ensure that patients were relaxed and comfortable during the session.
    - Before PFME, basic instructions of anatomy and physiology of micturition were given to patients.
    - The patients lied in supine position, knee bent, and feet placed on plinth.
    - Each patient was instructed on how to appropriately practice PFE while resting in the crock position with a neutral lumbar, including contracting PFM for a few seconds. The first step in performing PFM exercises was identifying PFM and performing the correct muscular contraction, which was then followed by both quick, gradual, and slow PFM contractions for improvement and rehabilitation. The exercises included (1) ten seconds of PFM elevation and (2) ten seconds of maintaining the muscles tight while counting to ten and repeating three sets of ten contractions (hold for 10 s and relax for 10 s). There will be a two-minute interval. Every three weeks of intervention, the training sessions will be progressed with three progressions (6 repetitions, sustained for 6 s of rest, 8 repetitions sustained for 8 s, 8 s of a break, and 10 repetitions, sustained for ten seconds, followed by a ten-second rest) [16].

### Sample size

This study is based on a sample of 45 patients with MS. G*Power was used to calculate the sample size at the start of the trial to reduce type 2 error. The preceding sample size was computed for the Mann-Whitney test to provide an effect size of 0.85, an actual test power of 0.79, and a 5% significance level on both sides. As a result, the projected sample size was 45. Data were examined for normality, homogeneity of variance, and the presence of extreme scores before statistical analysis. The boxplot is used to analyze the existence of extreme scores.

#### Statistical analysis

Statistical analysis was conducted using SPSS for Windows, version 18 (SPSS, Inc., Chicago, IL). Prior to final analysis, data were tested for normality assumption, homogeneity of variance, and presence of extreme scores. This exploration was done as a pre-requisite for parametric calculations of the analysis of difference.

Normality test of data using Shapiro-Wilk test and descriptive analysis using histograms with the normal distribution curve revealed that data was normally distributed for detrusor pressure at maximum flow rate, maximum cystometric capacity, detrusor pressure, and maximum flow rate. So, a paired sample t-test was used for within-group comparison (before and after treatment in the same group), and an ANOVA was used for between-group compression (between study groups A, B, and C before and after treatment). While the Shapiro-Wilk test and descriptive analysis using histograms with the normal distribution curve revealed that data was not normally distributed for bladder volume at the first desire to void, so nonparametric statistical tests in the form of Wilcoxon signed rank tests were used to compare pre- and post-treatment for each group, and the Kruskal-Wallis test was used to compare the two groups. Alpha level was set at 0.05.

## Results

The results of this study were presented under the following titles:

1. Descriptive Analysis of Subjects General Characteristics.
2. Analysis of the results of detrusor pressure at maximum flow rate.
3. Analysis of the results of Bladder volume at the first desire to void.
4. Analysis of the results of maximum cystometric capacity.
5. Analysis of the results of detrusor pressure.
6. Analysis of the results of the maximum flow rate.

### Subject’s general characteristics between groups

Statistically, there was no significant difference between both groups regarding age (*P*-value = 0.425). There was a non-significant difference in sex distribution between the three groups of the study (*P*-value = 0.407). There was no significant difference in type-MS distribution between the groups of the study. (*P*-value = 0.834) Table 1.

**Table 1:** Clinical characteristic of MS disease patients

Age (years)						
	Group A	Group B	Group C	Comparison		S
	Mean ± SD	Mean ± SD	Mean ± SD	F-value	*P*-value	
	38.20 ± 9.95	39.5 ± 8.15	34.3 ± 9.56	−0.855	0.436	NS
Sex distribution N (%)						
	Group A	Group B	Group C	Chi−square	***P***-value	
Female	12 (80%)	9 (60%)	9 (60%)	1.8	0.407	NS
Male	3 (20%)	6 (40%)	6 (40%)			
Types of MS distribution N (%)						
	Group A	Group B	Group C	Chi−square	***P***-value	
PPMS	1	2	3	1.459	0.834	NS
RRMS	12	11	11			
SPMS	2	2	1			
Total	15	15	15			

After treatment, the maximum flow rate increased significantly in groups A, B, and C with (*P*-value = 0.003), (*P*-value = 0.001), and (*P*-value = 0.008), respectively. But there was no significant difference in mean values of detrusor pressure at maximum flow rate after treatment between groups (A, B, and C) with a P-value of 0.781. And there was a significant increase in maximum cystometric capacity after treatment in groups A and C with (*P*-value < 0.001) and (P-value = 0.034), respectively. While there was no significant increase in maximum cystometric capacity after treatment in group B (*P*-value = 0.184). But there was a non-significant difference in mean values of maximum cystometric capacity after treatment between groups (A, B, and C) with a (*P*-value = 0.716). There was a significant increase in detrusor pressure after treatment in groups A, B, and C with (*P*-value = 0.014), (*P*-value = 0.078), and (*P*-value = 0.016), respectively. But there was a non-significant difference in mean values of detrusor pressure after treatment between groups (A, B, and C) with ( *P*-value = 0.730).

There was a significant increase in maximum flow rate after treatment in groups A and B with (*P*-value = 0.019) and (*P*-value = 0.016). While there was a non-significant increase in maximum flow rate after treatment (*P*-value = 0.052) in group C. Also, there was a non-significant difference in mean values of maximum flow rate after treatment between groups (A, B, and C) with (*P*-value = 0.218) (Tables 2 and 3).

**Table 2:** Comparison between results of all variables of urodynamic before and after treatment in study groups (A, B and C)

	Statistical tools	Group A		Group B		Group C	
		Pre treatment	Post treatment	Pre treatment	Post treatment	Pre treatment	Post treatment
Detrusor pressure at maximum flow rate	Mean ± SD	41.9 ± 28.27	57.2 ± 22.622	50.9 ± 37.078	66.4 ± 31.725	53.2 ± 37.988	63.8 ± 34.505
	Mean difference	−15.3		−15.5		−10.60000−	
	% of improvement	36.52%		30.45%		19.93%	
	T-value	−3.953		−4.653		−3.409−	
	P-value	0.003		0.001		0.008	
	Level of significance	Significant Increase		Significant Increase		Significant Increase	
Maximum cystometric capacity	Mean ± SD	543.4 ± 141.728	660.1 ± 111.105	538.7 ± 291.859	620.1 ± 159.931	471.4 ± 218.854	608 ± 166.982
	Mean difference	−116.7		−81.4		−136.6	
	% of improvement	21.48%		15.11%		28.98%	
	T-value	−7.066		−1.438		−2.49	
	P-value	0		0.184		0.034	
	Level of significance	Significant Increase		Non-significant Increase		Significant Increase	
Detrusor pressure	Mean ± SD	23 ± 17.487	31.2 ± 24.367	34.9 ± 22.073	39.9 ± 25.44	31.9 ± 22.418	35.3 ± 23.238
	Mean difference	−8.2		−5		−3.4	
	% of improvement	35.65%		14.33%		10.66%	
	T-value	−3.034		−1.987		−2.94	
	P-value	0.014		0.078		0.016	
	Level of significance	Significant Increase		Non-significant Increase		Significant Increase	
Maximum flow rate	Mean ± SD	7.65 ± 7.089	13.62 ± 6.359	6.83 ± 6.684	11 ± 6.375	7.09 ± 3.752	8.97 ± 4.489
	Mean difference	−5.97		−4.17		−1.88	
	% of improvement	78.04%		61.05%		26.52%	
	T-value	−2.852		−2.965		−2.233	
	*P*-value	0.019		0.016		0.052	
	Level of significance	Significant Increase		Significant Increase		Non-significant Increase	

**Table 3:** Results of ANOVA among the three groups

	Statistical tools		SS	MS	F-value	*P*-value	S
Detrusor pressure at maximum flow rate	Before treatment	Between Groups	713.267	356.633	0.296	0.746	NS
		Within Groups	32553.4	1205.681			
		Total	33266.667				
	After treatment	Between Groups	449.867	224.933	0.249	0.781	NS
		Within Groups	24379.6	902.948			
		Total	24829.467				
Maximum cystometric capacity	Before treatment	Between Groups	32451.267	16225.63	0.318	0.73	NS
		Within Groups	1378492.9	51055.29			
		Total	1410944.17				
	After treatment	Between Groups	14869.4	7434.7	0.339	0.716	NS
		Within Groups	592247.8	21935.1			
		Total	607117.2				
Detrusor pressure	Before treatment	Between Groups	766.067	383.033	0.887	0.424	NS
		Within Groups	11659.8	431.844			
		Total	12425.867				
	After treatment	Between Groups	378.867	189.433	0.319	0.73	NS
		Within Groups	16028.6	593.652			
		Total	16407.467				
Maximum flow rate	Before treatment	Between Groups	3.512	1.756	0.048	0.953	NS
		Within Groups	981.095	36.337			
		Total	984.607				
	After treatment	Between Groups	108.693	54.346	1.611	0.218	NS
		Within Groups	910.977	33.74			
		Total	1019.67				

The Kruskal-Wallis test revealed there was no significant difference in median values of bladder volume at the first desire to void before treatment between study groups (A, B, and C) with a *P*-value = 0.058). But in group A, the median of Bladder volume at the first desire to void after treatment was (2 ml), while in group B, the median of bladder volume at the first desire to void after treatment was (14 ml), and the median of bladder volume at the first desire to void in group C after treatment was (2 ml). Also, there was a significant difference in groups (A, B, and C) after treatment (*P*-value = 0.187) (Table 4**)**.

**Table 4:** Comparison between bladder volume at the first desire to void results before and after treatment in groups (A, B, and C)

	Statistical tools	Group A	Group B	Group C	Chi-Square	*P*-value	Sig.
Before treatment	Median	43	44	43.5	5.680	0.058	NS
After treatment	Median	55	63.5	58	3.357	0.187	NS
	Z-value	−2.805	−2.191	−2.803			
	Sig.	S	S	S			

## Discussion

The term ‘neurogenic detrusor overactivity (NDO)’ refers to a urodynamic finding that causes involuntary detrusor (bladder) contractions throughout the filling phase, which can be spontaneous or induced by an associated neurological condition. MS is one of the most widespread suprapontine disorders, causing NDO [17].

All urodynamic measurement measures improved statistically significantly within the groups (Groups A, B, and C) before and after treatment. Except for the maximal cystometric capacity in groups A and C, only group B showed a significant increase prior to and following treatment. However, there was no significant difference between the three groups.

This study is supported by [18], which indicates that in addition to PFM training, verbal feedback or device-assisted biofeedback for any type of urinary incontinence may be useful. However, that it was unclear if the higher advantage was attributable to the biofeedback or if it may be attributed to another difference between the groups under examination, such as a variance in contact time with the health specialist. Another study was shown combination PFM exercises with neuromadulation therapy improve PFM activity [19]. Biofeedback was shown to be the most effective therapy in a study of patients with Levator-Anis syndrome. Electrostimulation and massage were also employed. 87% of the biofeedback group, 45% of the electrostimulation group, and 22% of the massage group experienced adequate alleviation [20].

Kegel exercise produces increased strength in the levator ani muscle by enhancing support and decreasing ligament strain while increasing muscle activity [21]. Uroflowmetry measurements such as maximum flow rate, voided volume, voiding efficiency, total bladder capacity, voiding duration, and time to maximum flow rate increased considerably following PFMT therapy, according to this study [22].

Brusa et al. [23] investigated the potential influence of a 2-week course of low-frequency (1 Hz) TMS on LUT behavior in eight advanced Parkinson’s disease patients complaining of urine problems. TMS was able to temporarily improve LUT behavior in Parkinson’s disease patients, boosting bladder capacity and the initial sensation of filling phase, which lasted for up to 2 weeks after the stimulation ended [23]. This is the first randomized experiment combining PFMT and rTMS as therapy for neurogenic bladder dysfunction, as previously stated. PFMT has grown over decades as a first-line treatment for stress and mixed UI, with most studies indicating that it could enhance endurance and power of the muscles, as well as coordination and efficient bladder usage. It is a commonly suggested approach that combines behavioral science, nursing, and physiological muscle concepts [24]. rTMS had no impact, however the finding of a decrease in Pdet@Qmax appears to indicate greater urethral sphincter relaxation [25].

## Conclusions

Based on the findings of this study, it is possible to conclude that transcranial magnetic stimulation and EMG biofeedback had a beneficial effect on improving bladder function in patients with multiple sclerosis; therefore, it is recommended to provide a scientifically supported physical therapy program for patients with MS who have bladder problems. These approaches have a high level of safety and effectiveness, but EMG biofeedback has superiority.

## Supplementary Information

Below is the link to the electronic supplementary material.

ESM1 (DOCX 16.9 KB)

## Data Availability

Data will be held with the corresponding author and maybe available upon request.
