# Supplementary material for: Comparative study on the Impact of Transcranial Magnetic stimulation and Bio-feedback on overactive bladder in multiple sclerosis patients: a Randomized Clinical Trial
Source: Neurol Sci. 2024 Oct 21;46(3):1291–9. doi: 10.1007/s10072-024-07788-y (PMC11828820; doi:10.1007/s10072-024-07788-y)
Supplement: Supplementary file 1 — (DOCX 16.9 KB) [file 10072_2024_7788_MOESM1_ESM.docx]

**Neurological Sciences**

ID: NEUS-D-24-00273

•	Title: "Comparative Study on The Impact of Transcranial Magnetic stimulation and bio-Feedback on Overactive Bladder in Multiple Sclerosis Patients: A Randomized Clinical Trial"

Dear Editors,

We are providing a revised version of our paper following the comments and suggestions from the referees and also answering their questions. We have conducted a thorough revision of the paper following the received reviews. We want to thank the referees very much for the constructive criticisms which helped us to improve our paper. We also thank the editors for giving us the opportunity to revise our paper. Overall, we have included more detailed explanations and discussions. We also revised very carefully the presentation and organization of our paper to substantially improve clarity. Of course we have also fixed the typos and grammar issues with the help of native English speakers. We have included a word file for our paper with highlights text for easy identification of the changes made. This will hopefully help to clarify that the paper contains sufficient new material compared to the related work research, to merit publication in this post-conference special issue. Below we explain in more detail how we addressed the referees’ comments.

We gratefully thank the editor and all reviewers for their time spent making their constructive remarks and useful suggestions, which has significantly raised the quality of the manuscript and enabled us to improve the manuscript. Each suggested revision and comment, brought by the reviewers was accurately incorporated and considered.

Sincerely,

The Authors

**List of Responses to the Editor Comments**

| **Editor comments** | |
| --- | --- |
| **Editor Comment** | **Author Responses** |
| We have received the reports from our advisors on your manuscript, "Comparative Study on The Impact of Transcranial Magnetic stimulation and EMG bio-Feedback on Overactive Bladder in Multiple Sclerosis  Patients: A Randomized Clinical Trial", which you submitted to Neurological Sciences.Based on the advice received, I feel that your manuscript could be reconsidered for publication should you be prepared to incorporate major revisions. When preparing your revised manuscript, you are asked to carefully consider the reviewer comments which are attached, and submit a list of responses to the comments. If one or more reviewers have uploaded files related to their reviews, these files can be found online. | We acknowledge and appreciate the importance of maintaining the integrity of the peer review process. In our point-by-point response, we thoroughly evaluate the relevance of the suggested papers and include them in our study only if they contribute significantly to the context and content of our research. |

| **Reviewer #1** | |
| --- | --- |
| **Reviewer Comments** | **Authors Responses** |
| Were the patients also treated pharmacologically, including with cannabinoids, for their bladder disturbances? What DMTs were they taking to treat their MS? | Thank you very much for taking the time to provide such detailed feedback on our manuscript. We truly appreciate your kind words about our study.  All cases were recruited  depending on neurological and urological referrals; any patient who used drugs that affected urination during treatment was excluded from the study. |
| . Despite 12 tables are presented, details on EDSS, disease duration and other clinically relevant details are missing. | Thank you for your thoughtful comment. We have clarified it |
| At least half of the tables (12) and figures (6) are useless and should be removed. Descriptive statistics and other information could be easily incorporated in the main text, while presenting the results. | Thank you for your thoughtful comment, We removed figures and rejustified tables and described it statistically. |
| A more detailed description of the TMS and bio-feedback approaches need to be presented. | thank you for your thoughtful comment, we have  clarified it |
| The first description of TMS effects on bladder symptoms in MS should be quoted and properly discussed (Centonze D et al., Mult Scler 2007 Mar;13(2):269-71), also considering the different stimulation protocols adopted and different results. | Thank you very much for taking the time to provide such detailed feedback on our manuscript, we added that |
| Name of the EC that approved the study, and the approval number must be provided. | We added the name of EC in Methods, but it is blind for review process |
| English language needs extensive revision and some typos should be corrected (for example TNS instead of TMS at page 2. | Thank you for bringing this to our attention, We have addressed the issues related to writing style, grammar, organization, and formatting. |
